# *Anaplasma phagocytophilum *infection in a domestic cat in Finland: Case report

**DOI:** 10.1186/1751-0147-52-62

**Published:** 2010-11-15

**Authors:** Helka M Heikkilä, Anna Bondarenko, Andrea Mihalkov, Kurt Pfister, Thomas Spillmann

**Affiliations:** 1 Department of Equine and Small Animal Medicine, Faculty of Veterinary Medicine, P.O. BOX 57, 00014 University of Helsinki, Helsinki, Finland; 2 Institute of Comparative Tropical Medicine and Parasitology, Leopoldstr. 5, 80802 Munich, Germany

## Abstract

**Background:**

Anaplasmosis is a vectorborne disease caused by the gram-negative bacterium *Anaplasma phagocytophilum*. This species displays positive tropism to granulocytes and can cause illness in several mammalian species, including cats, dogs, and humans. It is considered as an emerging disease in Europe. The clinical signs are nonspecific and include fever, lethargy, and inappetence. The most typical hematologic abnormality is thrombocytopenia. A tentative diagnosis can be made by detecting intracytoplasmic morulae inside neutrophils. The diagnosis is confirmed by PCR and serology in paired serum samples. A sample for PCR analysis should be taken before treatment. Anaplasmosis is treated with doxycycline.

**Case presentation:**

A feline case of anaplasmosis is presented. The history, clinical presentation, diagnostics, treatment, and follow-up are discussed.

**Conclusions:**

This case indicates that *Anaplasma phagocytophilum *infects cats in Finland. To provide accurate treatment, anaplasmosis should be listed as a differential diagnosis in cats suffering from acute febrile illness with previous tick exposure.

## Background

Anaplasmosis is a vectorborne disease caused by the gram-negative bacterium *Anaplasma phagocytophilum *(encompassing the former *Ehrlichia phagocytophila, Ehrlichia equi*, and the human granulocytic ehrlichiosis agent) [1]. Although anaplasmosis has been known for decades as a tickborne fever affecting domestic ruminants in Europe [2], *A. phagocytophilum *can also infect several other mammalian species, including dogs [3], horses [4], cats [5], small rodents [6], cervids [6-9], European bison [2], wild boars, red foxes, European hares [9], donkeys [10], and humans [11].

The bacterium has been detected in almost all European countries [2,12]. In Finland *A. phagocytophilum *infection has previously been reported only in cattle [13]. This is the first reported case of anaplasmosis in a cat as well as in any companion animal in Finland.

## Case presentation

### History

A 3.5-year-old sterilized female Maine coon cat was brought to the Veterinary Teaching Hospital of Helsinki University with lethargy and poor appetite in July 2008. The cat had symptoms for 3 days, including hiding, ocular discharge, and not drinking, urinating, or defecating during that period. The symptoms began immediately after returning from a summer cottage in southeastern Finland, where the cat was often kept outdoors on a leash. The owner noticed two ticks attached to the cat's skin, 1 day before the appointment. The cat was previously healthy except for suspected endometriosis treated with ovariohysterectomy 1 year before.

### Clinical presentation

The abnormal findings in the clinical examination were fever (39.5°C), tachypnea, bilaterally increased lung sounds, slightly painful cranial abdomen, and discharge in both eyes. Additionally, the cat had four skin lesions, indicating previous tick exposure. One *Ixodes ricinus *tick was found attached.

### Diagnostics

Thoracic and abdominal radiographs were obtained. Venous blood sample was taken into EDTA and heparin tubes. The complete blood count and biochemistry profile were analyzed. The radiographs showed no abnormal changes except for minimal bronchial pattern in the lungs. Lymphopenia (lymphocytes 0.4 × 10^9^/L; reference range 1.3 - 7.5 × 10^9^/L) and mild hyperglycemia (glucose 7.7 mmol/L; reference range 3.5 - 5.7 mmol/L) were identified as the only abnormalities in the blood sample. However, when the blood smear was analyzed, we detected intracytoplasmic morulae in the neutrophils. After viewing the morulae, we confirmed the diagnosis of anaplasmosis by PCR analysis at the laboratory of the Institute of Comparative Tropical Medicine and Parasitology in Munich, Germany. The sample was tested by a modified real-time PCR [14] targeting the msp2 gene of *A. phagocytophilum *(primers msp25 [5'TTATGATTAGGCCTTTGGGCATG -3'] and msp23 [5'-TCAGAAAGATACACGTGCGCCC-3'] [15]). The reaction was carried out in a BioRad iCycler iQ (BioRad, Munich, Germany) under cycling conditions comprising an initial activation (95°C, 15 min), followed by 50 cycles denaturation (94°C, 15 s), and annealing-extension (60°C, 60 s). DNA of an infected dog and sterile water were used as a positive and negative control, respectively.

### Treatment

Initially, the cat was given subcutaneous injections of amoxicillin clavulanic acid (14 mg/kg), metoclopramide (0.4 mg/kg), ranitidine (2.1 mg/kg), meloxicam (0.3 mg/kg), and subcutaneous fluid therapy with Ringer's lactate. The treatment was changed to oral doxycycline (8, 4 mg/kg twice daily for 30 days) the next day after finding the morulae and with improved appetence of the cat. Percutaneous fipronil (8.4 mg/kg) was prescribed for once monthly administration to prevent further tick infestation.

### Follow-up

At the control visit 30 days after initiating the treatment, the owner reported that the cat was clinically healthy. There were no abnormal findings in clinical examination, except for tachypnea. A serum biochemistry profile and complete blood count were obtained. The *A. phagocytophilum *antibody titer was analyzed. No significant hematological or biochemical abnormalities were detected; lymphocytes were also within the reference range. The *A. phagocytophilum *IgG antibody titer was > 1:128. A titer of 1:32 or more was regarded as positive.

## Discussion

*Anaplasma phagocytophilum *is transmitted by various ticks, *Ixodes ricinus *being the vector in Europe [1,12,16,17]. Transovarial transmission in ticks does not occur [1,18] and the pathogen is maintained in the environment by a wide range of hosts such as grazing domestic ruminants, wild rodents [18], and cervids [19]. There is some evidence that migratory birds may facilitate the dispersal of infected ticks to new regions [20]. The prevalence of *A. phagocytophilum *infection has not been studied in competent reservoir animals or birds in Finland, but the pathogen was recently found in *Ixodes ricinus *ticks from the southeastern part of the country (E. Hasu, unpublished data). Interestingly, the present cat had been outdoors in the same region. *Anaplasma phagocytophilum *has previously been detected in ticks in all of the neighboring countries; Estonia [21], European Russia [16], Sweden [22], and Norway [23].

The clinical manifestation of anaplasmosis varies widely and is believed to be at least partially host-dependent [24,25]. In a recent study of experimental anaplasmosis in sheep, the severity of the clinical manifestation also varied among different strains of the bacteria [25]. The clinical signs of anaplasmosis in cats resemble those of the disease in dogs. In both species the most common manifestation is an acute febrile illness with lethargy and inappetence [5,17,24,26], as shown here. The cat also showed other nonspecific symptoms. Various other symptoms have been reported in cats, including tachypnea, hyperesthesia, muscle and joint pain, lameness, neck rigidity, lymphadenomegaly, gingivitis, periodontitis, conjunctivitis, weight loss, vomiting, pharyngitis, polydipsia, hematuria, and neurological problems such as tremors, incoordination, and shyness [27]. Anaplasmosis causes immunosuppression [27,28], and some of the many symptoms reported in cats may have been caused by opportunistic infections. The present cat showed tachypnea with mild bronchial pattern in the lungs. Tachypnea persisted after treatment and may have been caused by the stress involved in examination, since there was no evidence for a pulmonary infection.

**Figure 1:**
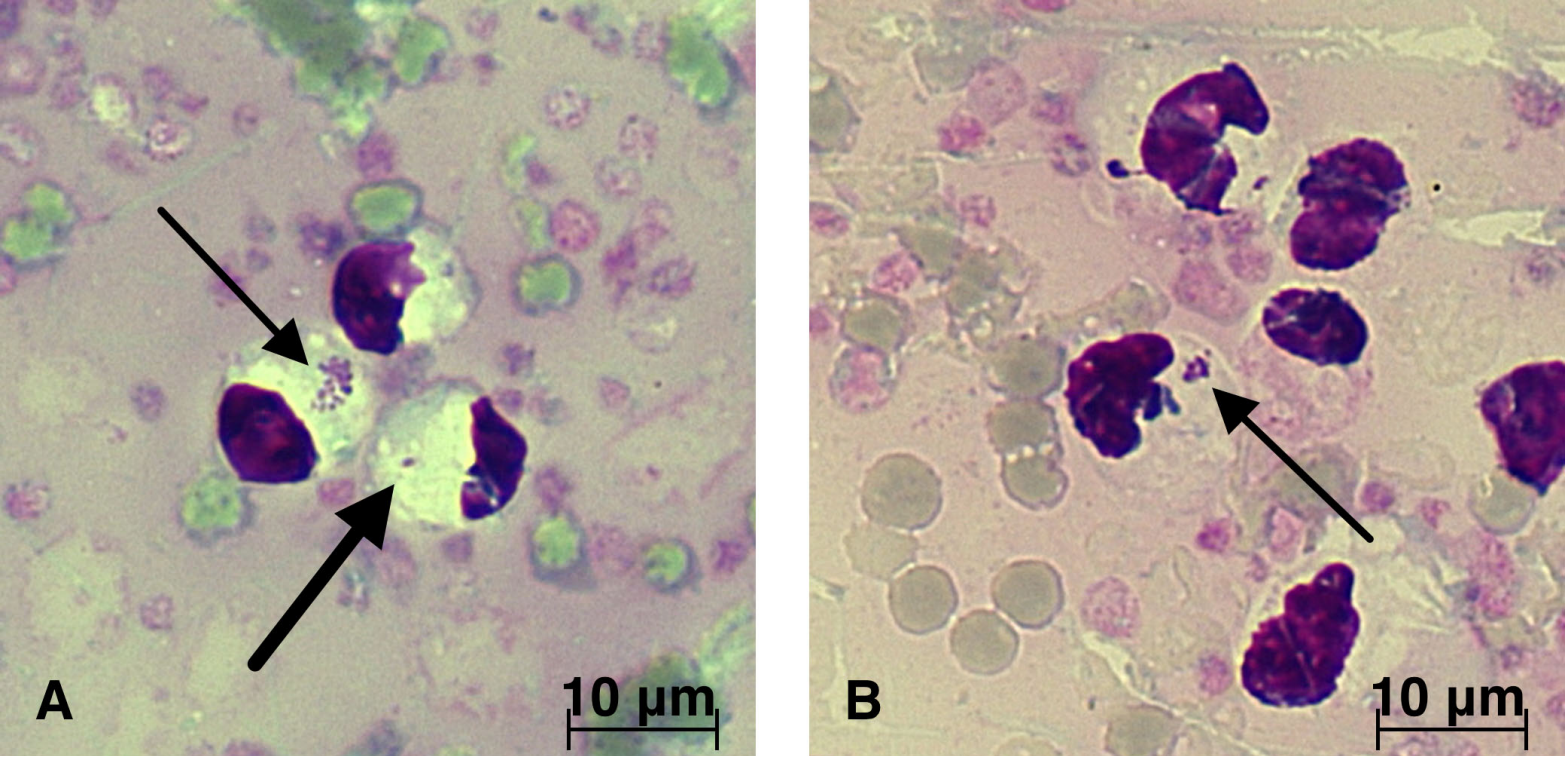
**Microscopic evidence for *A. phagocytophilum *infection in a cat**. The arrows in A and B indicate the ehrlichia-like inclusions inside the neutrophilic granulocytes in a cat with anaplasmosis. The inclusions can appear as single (thick arrow) or as colonies (morulae) in a cytoplasmic vacuole. Peripheral blood smear, May-Grünwald-Giemsa, original magnification × 1000.

The most commonly reported hematologic abnormality in anaplasmosis is thrombocytopenia [5,17,24,29], which is assumed to be caused by destruction of the platelets [30]. Thrombocytopenia is mild to moderate in cats [5,17] and moderate to severe (< 50 × 10^9^/L) in dogs [29]. The cat presented here did not have thrombocytopenia, but lymphopenia, which is consistent with previous reports of feline anaplasmosis. Other reported hematologic abnormalities include neutrophilia with left shift and anemia. The combination of different hematological abnormalities varies [5,17,27,28] and is most probably related to the duration of infection prior to sampling. Monoclonal gammopathy and hyperglycemia were reported as serum biochemical abnormalities in feline anaplasmosis [5,17,24,27,29]. The present cat had hyperglycemia. As discussed in previous reports, this was likely attributed to stress. The nonspecific symptoms of feline anaplasmosis can resemble a wide range of other diseases, including other systemic infections, immune-mediated diseases, and neoplasia. As in the present case, a tentative diagnosis of anaplasmosis can be made by direct microscopic examination of May-Grünwald-Giemsa-, Wright-, or Romanowsky-stained peripheral blood smears. The bacterium appears as a single inclusion with a diameter of 0.54 - 1.3 microns, or as colonies (morulae) of 1.5 - 5.0 microns in the cytoplasm of infected neutrophilic and eosinophilic granulocytes [27,30-32] (Figure 1). In feline experimental infection with *A. phagocytophilum*, intracytoplasmic inclusions appeared in neutrophils in 7 - 9 days [28]. The number of infected cells varies from 1% to 24% in different reports. The morulae can be detected only in the acute stage of the disease [5,27], without pre-treatment with antibiotics [31]. No infected cells were found in the peripheral blood smear of the present cat after a 30-day course of doxycycline. In the first reported feline case of anaplasmosis in Sweden in 1999, the previously infected cells could no longer be identified after 15 days of antibiotic therapy [5].

Antibodies against *A. phagocytophilum *can be examined by IFA test [17,33], ELISA [10,34], or western immunoblot assay [35]. In experimentally infected cats, seroconversion occurred at the earliest within 14 days after infection [28]. At the control visit, the present cat showed positive antibody titer despite doxycycline therapy. Various case reports showed that all cats seroconverted in the course of the disease and that treatment with antibiotics did not prevent the seroconversion [5,17,36]. Antibody titers can remain high during the months after infection regardless of antibiotic treatment [5,17].

Interpretation of serological results can be confusing, due to seronegativity in the acute stage of the disease, persisting high antibody titers, and possible cross-reactivity with antibodies to other ehrlichial bacteria. To verify the infection by serology only, both acute and convalescent serum samples should be examined [35]. In humans and dogs, a 4-fold increase in the antibody titer should be detected in 4 weeks [35,37]. Evaluation of the evidence for cross-reactivity between *A. phagocytophilum *and other ehrlichial bacteria in cats is confusing, due to reclassification of the genera in 2001 [1,38]. In cases of feline anaplasmosis in which the diagnosis was confirmed by PCR, no cross-reactivity was detected with *Ehrlichia canis *and *Neorickettsia risticii *[5,17]. However, although uncommon, cross-reactivity exists in dogs [39]. Cross-reactivity with antibodies to the more closely related bacterium *Anaplasma platys *is likely [40,41], but there is only limited evidence of feline *A. platys *infection [35].

PCR analysis is a direct and reliable method for confirming the diagnosis in the acute stage of anaplasmosis [42]. The analysis is most often made from peripheral blood samples, but bone marrow or splenic tissue can also be used [30]. Samples for PCR analysis should be taken before treatment [35]. In cats experimentally infected with *A. phagocytophilum*, DNA could be detected from blood and bone marrow from 7 to 11 days after infection [28].

Most of the numerous PCR assays developed for the detection of *A. phagocytophilum *use the 16 S rRNA or the outer surface protein msp2 genes as targets [30]. The sensitivities and specificities vary among the different assays and primers; some also detect DNA from other closely related bacteria [30,43].

*Anaplasma phagocytophilum *is resistant to various antibiotics, including beta-lactam and macrolide compounds, sulfonamides, lincosamides, carbapenem antibiotics, and aminoglycosides [44-47]. In addition, its sensitivity to chloramphenicol is poor [44,45,48]. Tetracyclines, mainly doxycycline, are considered to be the antibiotics of choice for treatment [39,47]. Rifampin and fluoroquinolones are effective against *A. phagocytophilum in vitro *[45-47]. Rifampin led to clinical cure in human anaplasmosis [49,50], while relapse was reported after fluoroquinolone therapy [51].

Oral doxycycline 10 mg/kg/day for 20 - 30 days and oral tetracycline 22 mg/kg every 8 h for 21 days can be used to successfully treat anaplasmosis in cats [5,17]. Doxycycline should be given with water or food to prevent esophageal strictures [52]. If the cat is anorectic, antibiotic treatment can be started intravenously [5,17], but phlebitis was reported after intravenous doxycycline administration [17]. In acute illness the response to treatment is rapid and seen in 24 - 48 hours [17,39]. The owner of the present cat reported that the symptoms resolved in a few days. However, infection may persist for months despite treatment, and treatment with tetracyclines, even for 30 days, may not be adequate for elimination of the pathogen in cats. Conversely, complete recovery without antimicrobial treatment was reported [17]. In murine experimental studies and in a natural case of human anaplasmosis, previous infection with *A. phagocytophilum *did not prevent infection. However, the clinical course of the disease can be milder and of shorter duration [53,54].

The prevalence of anaplasmosis is increasing in both animals and humans [30,55]. The increase may be a result of several factors, including growing awareness of vectorborne diseases and how global climate change affects the distribution and prevalence of reservoir animals and vectors. Despite the increased prevalence of anaplasmosis in general, feline anaplasmosis is infrequently diagnosed. Although this can partly be related to the rapid elimination of ticks due to delicate grooming [56], the pathogenicity of tickborne pathogens in cats requires further research.

## Conclusions

This feline case is the first reported *A. phagocytophilum *infection in any companion animal in Finland. *Anaplasma phagocytophilum *infection should be considered as a differential diagnosis in cats suffering from an acute febrile illness with previous tick exposure.

## Consent

Written informed consent was obtained from the owner for publication of this case report. A copy of the written consent is available for review by the Editor-in-Chief of this journal.

## Competing interests

The authors declare that they have no competing interests.

## Authors' contributions

HMH carried out the clinical examination and treatment of the present cat and is the main author of this paper. AB and AM carried out the molecular diagnostics, while AB provided the pictures, and assisted to draft the manuscript. KP and TS reviewed the draft and helped to write the final version. All authors have read and approved the final manuscript.
